# Multi-omics integration and Mendelian randomization elucidate the PARP16–UPR axis driving chemoresistancein gastric cancer

**DOI:** 10.3389/fonc.2026.1785100

**Published:** 2026-05-01

**Authors:** Shuchang Tao, Hui Yang

**Affiliations:** Anhui University of Traditional Chinese Medicine First Affiliated Hospital, Hefei, Anhui, China

**Keywords:** cancer stem cells, cisplatin resistance, gastric cancer, Mendelian randomization, multi-omics, PARP16, therapeutic target, unfolded protein response

## Abstract

**Background:**

Acquired resistance to cisplatin-based chemotherapy is common in patients with gastric cancer (GC) and significantly limits treatment efficacy. The aim of this study was to investigate molecular features associated with GC chemoresistance using an integrative multi-level analytical framework combined with Mendelian randomization (MR), followed by cellular validation of key candidates.

**Methods:**

Transcriptome datasets GSE14210 and GSE31811 were obtained from the Gene Expression Omnibus (GEO) database to identify differentially expressed genes (DEGs), followed by Gene Ontology (GO) and Kyoto Encyclopedia of Genes and Genomes (KEGG) enrichment analyses to explore potential pathways. A total of 113 machine learning model combinations were applied for feature selection. MR analysis integrating expression quantitative trait loci (eQTLs) and genome-wide association study (GWAS) data was conducted to assess causal relationships between candidate genes and chemoresistance. The single-cell dataset GSE183904 was used to examine cell-type-specific expression patterns. Cisplatin-resistant NCI-N87/DDP cells were then established *in vitro*, and qRT-PCR, Western blotting, and drug sensitivity assays were performed to evaluate gene expression and function. Pathway inhibitors were applied to test the reversal of resistance.

**Results:**

A total of 827 DEGs were identified, mainly enriched in immune response, ECM interactions, metabolic reprogramming, and signaling pathways such as PI3K–Akt and MAPK. Among the machine learning models, the Stepglm[both] + Random Forest (RF) model achieved the best performance [area under the curve (AUC) = 0.865] and identified several core candidate genes. MR analysis supported potential risk associations for TRABD, RXRA, DEFA4, PARP16, SLC12A9, and TMEM132A, with PARP16 consistently highlighted across transcriptomic, machine learning, and MR analyses. *In vitro* experiments showed that PARP16 expression was elevated by approximately 3.1-fold in NCI-N87/DDP cells, accompanied by activation of the unfolded protein response (UPR) and suppression of apoptosis, and an elevated cisplatin IC_50_ of 11.82 μg/mL. Inhibition of the PARP16–UPR axis significantly reduced the IC_50_ to 4.67 μg/mL and restored DNA damage and apoptosis, demonstrating synergistic effects.

**Conclusions:**

PARP16 emerged as a key candidate associated with chemoresistance in GC. Its elevated expression in stem-like cell populations and resistant cell models was associated with UPR activation, and targeting the PARP16–UPR axis restored cisplatin sensitivity. Targeting the PARP16–UPR axis effectively reverses resistance, providing new insights and potential therapeutic strategies for overcoming chemoresistance in GC.

## Introduction

Gastric cancer (GC) remains one of the most prevalent and lethal malignancies of the digestive system worldwide, imposing a particularly heavy disease burden in East Asia (1–3). For patients with advanced GC, systemic chemotherapy remains a central therapeutic strategy, with cisplatin (DDP)-based regimens widely adopted in clinical practice (4–7). However, the majority of patients eventually develop acquired chemoresistance, leading to diminished therapeutic efficacy and high recurrence rates (8–10). Current evidence suggests that GC chemoresistance arises from a complex interplay of molecular alterations, including dysregulated drug transport, enhanced DNA damage repair, suppression of apoptotic signaling, metabolic reprogramming, and remodeling of the tumor microenvironment (11–14). The molecular basis of these processes is complex and remains incompletely understood.

Accumulating studies have highlighted the role of stress-adaptive signaling pathways in promoting tumor cell survival under chemotherapeutic stress. Among these, activation of endoplasmic reticulum (ER) stress-associated unfolded protein response (UPR) signaling has been repeatedly linked to chemotherapy resistance across multiple cancer types (15–17). The PERK- and IRE1-mediated branches of the UPR contribute to the maintenance of proteostasis and cellular homeostasis, enabling cancer cells to withstand chemotherapy-induced apoptosis (18–20). However, the upstream regulators responsible for sustained UPR activation in GC remain poorly defined.

With the rapid development of high-throughput sequencing technologies, multi-omics approaches such as transcriptomics and single-cell sequencing have provided valuable resources for elucidating the mechanisms of GC resistance (21–23). While these strategies have generated large-scale datasets and numerous candidate genes, most findings are derived from correlation-based analyses, limiting their ability to distinguish causal regulators from secondary or compensatory changes. Mendelian randomization (MR), a genetic epidemiological method based on germline variants, provides a complementary framework for causal inference (24–26). By integrating expression quantitative trait loci (eQTL) and genome-wide association study (GWAS) data, MR can overcome confounding and reverse causation, thereby improving the reliability of identifying true resistance-associated genes (27, 28).

In this context, our integrative multi-level analysis combined transcriptomic profiling, machine learning, MR, and single-cell validation to prioritize candidate genes associated with cisplatin resistance in GC. Single-cell transcriptomic data further revealed that PARP16 expression was enriched in tissue stem cell populations within tumors, suggesting a potential link to cancer stemness and adaptive resistance. However, the functional role and mechanistic contributions of PARP16 in GC chemoresistance remain poorly defined.

In the present study, bulk transcriptomic datasets (GSE14210 and GSE31811), single-cell sequencing data (GSE183904), machine learning-based feature selection, and MR analysis were integrated to systematically identify genes associated with cisplatin resistance in GC. A cisplatin-resistant GC cell model (NCI-N87/DDP) was subsequently established to validate the biological function of PARP16. By examining its involvement in ER stress signaling and UPR activation, this study aimed to clarify the role of PARP16 in chemoresistance and to explore the therapeutic potential of targeting the PARP16–UPR axis to restore cisplatin sensitivity. Collectively, these findings provide mechanistic insight into GC chemoresistance and support PARP16 as a potential biomarker and therapeutic target.

## Materials and methods

### Data acquisition and preprocessing

**Figure 1:**
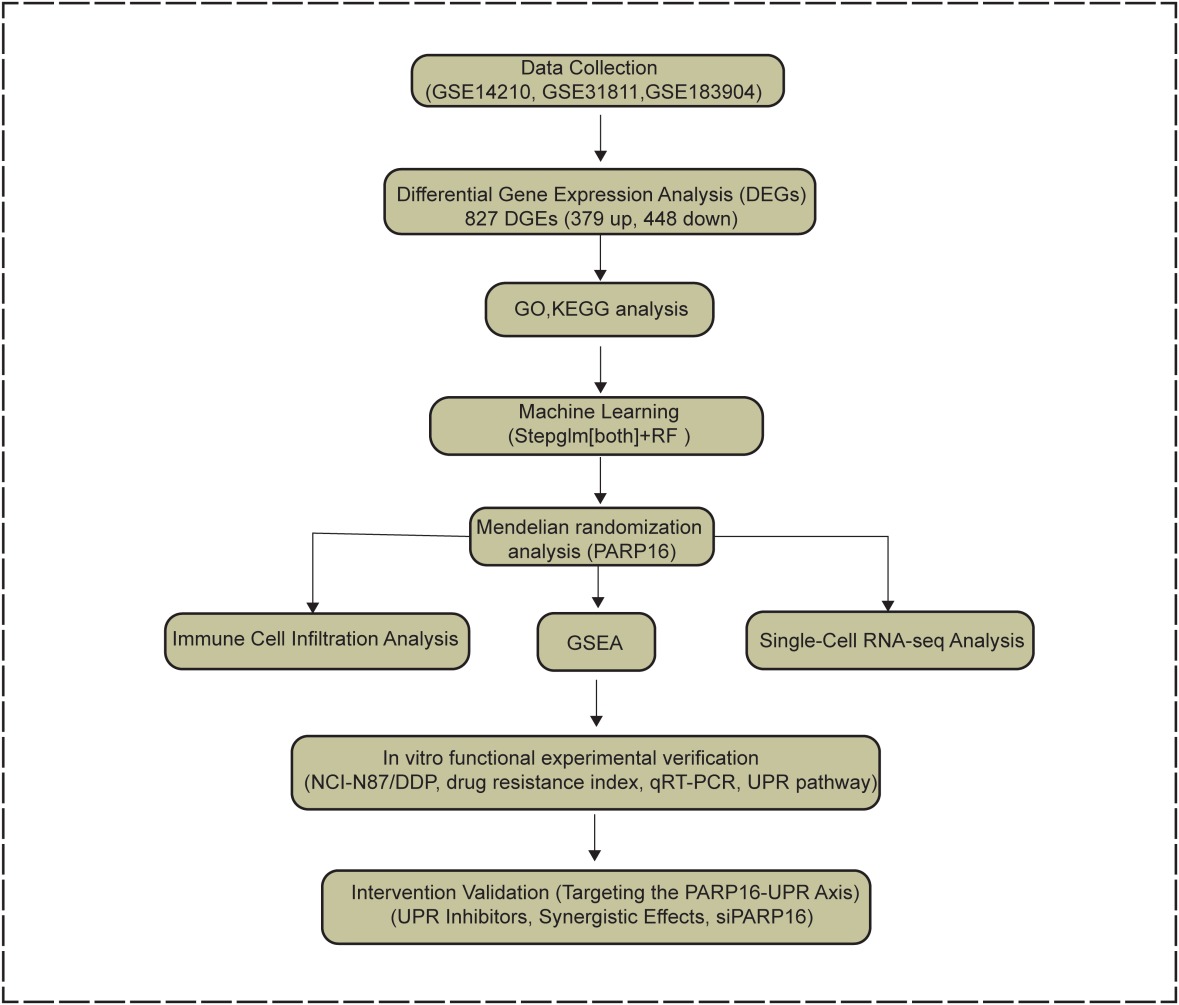
Flowchart of this study. This study employed a multi-level integrative analytical framework to identify key regulators of cisplatin resistance in gastric cancer. Transcriptomic datasets (GSE14210 and GSE31811) were used to identify differentially expressed genes associated with chemoresistance, followed by GO and KEGG enrichment analyses. Machine-learning-based feature selection was applied to prioritize candidate genes. MR analysis integrating eQTL and GWAS datasets was used to provide genetically supported associations, and single-cell RNA sequencing data (GSE183904) were analyzed to define the cellular context of PARP16 expression. Functional experiments in cisplatin-resistant GC cell models were then performed to validate the role of the PARP16–UPR axis in mediating chemoresistance.

Transcriptome datasets GSE14210 and GSE31811 related to GC chemoresistance were retrieved from the Gene Expression Omnibus (GEO) database, together with the single-cell transcriptomic dataset GSE183904. Raw data were processed in R (v4.2.2) using the limma package for background correction, normalization, and batch effect removal. Differentially expressed genes (DEGs) were identified with thresholds of |log2FC| > 0.263 and a false discovery rate (FDR) < 0.05 (29, 30). The overall study workflow is shown in **Figure 1**. Briefly, this study adopted a multi-level integrative analytical framework in which transcriptomic differential analysis was followed by machine learning prioritization, MR-based genetic support, single-cell contextual validation, and experimental verification.

### Differential expression and functional enrichment analysis

Based on the identified DEGs, we performed Gene Ontology (GO; Biological Process, Cellular Component, and Molecular Function) and Kyoto Encyclopedia of Genes and Genomes (KEGG) pathway enrichment analyses using the clusterProfiler package (31), with FDR < 0.05 considered statistically significant. Enrichment results were visualized using volcano plots, heatmaps, and bubble plots to characterize biological processes and signaling pathways potentially involved in GC chemoresistance.

### Machine learning modeling and core gene selection

A total of 113 machine learning model combinations were constructed to further refine candidate genes, including Least Absolute Shrinkage and Selection Operator (LASSO), Elastic Net, Stepwise Generalized Linear Model (GLM), Random Forest (RF), Gradient Boosting Machine (GBM), and Support Vector Machine (SVM). Tenfold cross-validation was used to evaluate performance, comparing classification ability in both training and validation cohorts. Among these models, the Stepglm[both] + RF combination achieved the best performance [training area under the curve (AUC) = 0.954; validation AUC = 0.865]. Genes selected by this optimal model were subsequently defined as core candidates associated with chemoresistance.

### Mendelian randomization analysis

MR analysis was conducted using the TwoSampleMR framework to assess potential causal relationships between candidate gene expression and GC chemoresistance (32, 33). Exposure data were obtained from publicly available eQTL databases [Genotype-Tissue Expression (GTEx) and eQTLGen], which capture genetic variants that regulate gene expression levels rather than alter protein function. These eQTL-derived single-nucleotide polymorphisms (SNPs) primarily reside in regulatory regions (promoters, enhancers, and UTRs) and modulate mRNA transcription. Outcome data were obtained from GWAS datasets (FinnGen, IEU OpenGWAS) (34). SNPs significantly associated with candidate genes were selected as instrumental variables, followed by LD pruning (*r*² < 0.01, window 10,000 kb). The inverse variance weighted (IVW) method was used as the primary estimator of causal effect, with MR-Egger, weighted median, and leave-one-out approaches applied for sensitivity analyses. Results were presented as odds ratios (ORs) with 95% confidence intervals (CIs), and *p* < 0.05 was considered statistically significant. MR analysis based on publicly available eQTL and GWAS summary statistics was performed to evaluate genetically supported associations, with sensitivity analyses used to assess pleiotropy and robustness.

*F* statistics were calculated for each SNP to evaluate instrument strength, and SNPs with adequate instrument strength were retained to minimize weak-instrument bias. Horizontal pleiotropy was assessed using the MR-Egger intercept test, and heterogeneity among SNP instruments was evaluated using Cochran’s *Q* statistics.

### Immune infiltration analysis

CIBERSORT was applied to estimate the relative proportions of 22 immune cell subpopulations in GC tissues (35). Correlation analyses between PARP16 expression and immune cell infiltration were further conducted using Spearman’s correlation test, with *p* < 0.05 considered significant.

### Gene set enrichment analysis

Samples were stratified into high- and low-expression groups according to PARP16 expression levels. Gene set enrichment analysis (GSEA) was performed using the clusterProfiler package, with thresholds of *p* < 0.05, |NES| > 1, and FDR < 0.25 (36). Enrichment results were visualized with the enrichplot package to explore pathway differences in energy metabolism, protein synthesis, and immune-related signaling between groups.

### Single-cell transcriptome analysis

Using the GSE183904 dataset, single-cell analysis was performed in Seurat (v4.3.0), including quality control, normalization, PCA, UMAP dimensionality reduction, and cell-type annotation (37). Marker genes were used for cluster identification, and PARP16 expression was compared between GC and normal tissues across different cell types, with particular focus on enrichment in tissue stem cells.

### Cell lines and establishment of resistant sublines

The human GC cell line NCI-N87 was obtained from the Cell Bank of the Chinese Academy of Sciences (Shanghai, China). Cells were cultured in RPMI-1640 medium supplemented with 10% fetal bovine serum (FBS) in a humidified incubator at 37 °C with 5% CO_2_. The cisplatin-resistant subline NCI-N87/DDP was established in our laboratory by exposing parental NCI-N87 cells to gradually increasing concentrations of cisplatin (0.2–2 μg/mL) over an extended culture period. Cells with stable resistance were maintained in culture medium containing 1 μg/mL cisplatin. The cisplatin-resistant GC cell line AGS/DDP was commercially obtained from Shanghai FUYUBIO Biotechnology Co., Ltd. (Shanghai, China). Cell line identity was authenticated by short tandem repeat (STR) profiling, and mycoplasma contamination was confirmed to be negative. AGS/DDP cells were cultured in RPMI-1640 medium supplemented with 10% FBS under standard conditions (37°C, 5% CO_2_) according to the supplier’s instructions. To maintain the resistant phenotype, both NCI-N87/DDP and AGS/DDP cells were routinely cultured in medium containing low-dose cisplatin and transferred to drug-free medium for at least 72 h prior to subsequent experiments.

### RNA extraction and qRT-PCR

Total RNA was extracted using TRIzol reagent (Invitrogen) and reverse transcribed into cDNA with the PrimeScript RT Kit (Takara). Gene expression was quantified by SYBR Green-based real-time PCR on an ABI ViiA 7 system, with GAPDH as an internal control. Relative expression levels were calculated using the 2^−ΔΔCt^ method. Target genes included PARP16, BiP, PERK, XBP1s, CHOP, CASP3, and BAX.

### Cell viability and drug sensitivity assays

Cell sensitivity to cisplatin was assessed using the Cell Counting Kit-8 (CCK-8) assay. NCI-N87 and NCI-N87/DDP cells were seeded in 96-well plates at 5,000 cells/well and treated with varying concentrations of cisplatin (1–32 μg/mL) for 48 h. Absorbance at 450 nm was measured, and dose–response curves were plotted to calculate the half-maximal inhibitory concentration (IC_50_).

### Pathway inhibition and synergy analysis

NCI-N87/DDP cells were treated with the PERK inhibitor GSK2606414 in combination with cisplatin to examine the involvement of the PARP16–UPR axis in cisplatin resistance. Cell viability was determined by CCK-8 assay, and drug interactions were assessed using CompuSyn software to calculate the combination index (CI), with CI < 1 indicating synergy.

### Protein extraction and Western blot

Cells subjected to different treatments were lysed with RIPA buffer, and protein concentration was quantified using the bicinchoninic acid (BCA) assay. Equal amounts of protein (30 μg) were separated by SDS-PAGE and transferred to membranes. After blocking with 5% bovine serum albumin (BSA) for 1.5 h, membranes were incubated overnight with primary antibodies against PARP16, BiP, PERK, p-PERK, cleaved caspase-3, γ-H2AX, and GAPDH, followed by horseradish peroxidase (HRP)-conjugated secondary antibodies for 1.5 h. Bands were visualized using an enhanced chemiluminescence (ECL) detection system, and densitometry was performed with ImageJ, normalized to GAPDH. Western blot analysis was also performed in AGS/DDP cells under the same experimental conditions using the same set of antibodies, to validate changes in PARP16, BiP, p-PERK, cleaved caspase-3, and γ-H2AX expression (**Supplementary Figure 1**).

### Statistical analysis

All experiments were independently repeated at least three times. Results were expressed as mean ± standard deviation (SD). Comparisons between two groups were performed using independent-sample *t*-tests, while multiple-group comparisons were assessed with one-way analysis of variance (ANOVA). ROC curves were used to evaluate model performance, and Spearman’s method was applied for correlation analysis. Statistical analyses were conducted using SPSS 22.0 and GraphPad Prism 9.0, with *p* < 0.05 considered statistically significant.

## Results

### Identification of chemoresistance-related genes in gastric cancer

**Figure 2:**
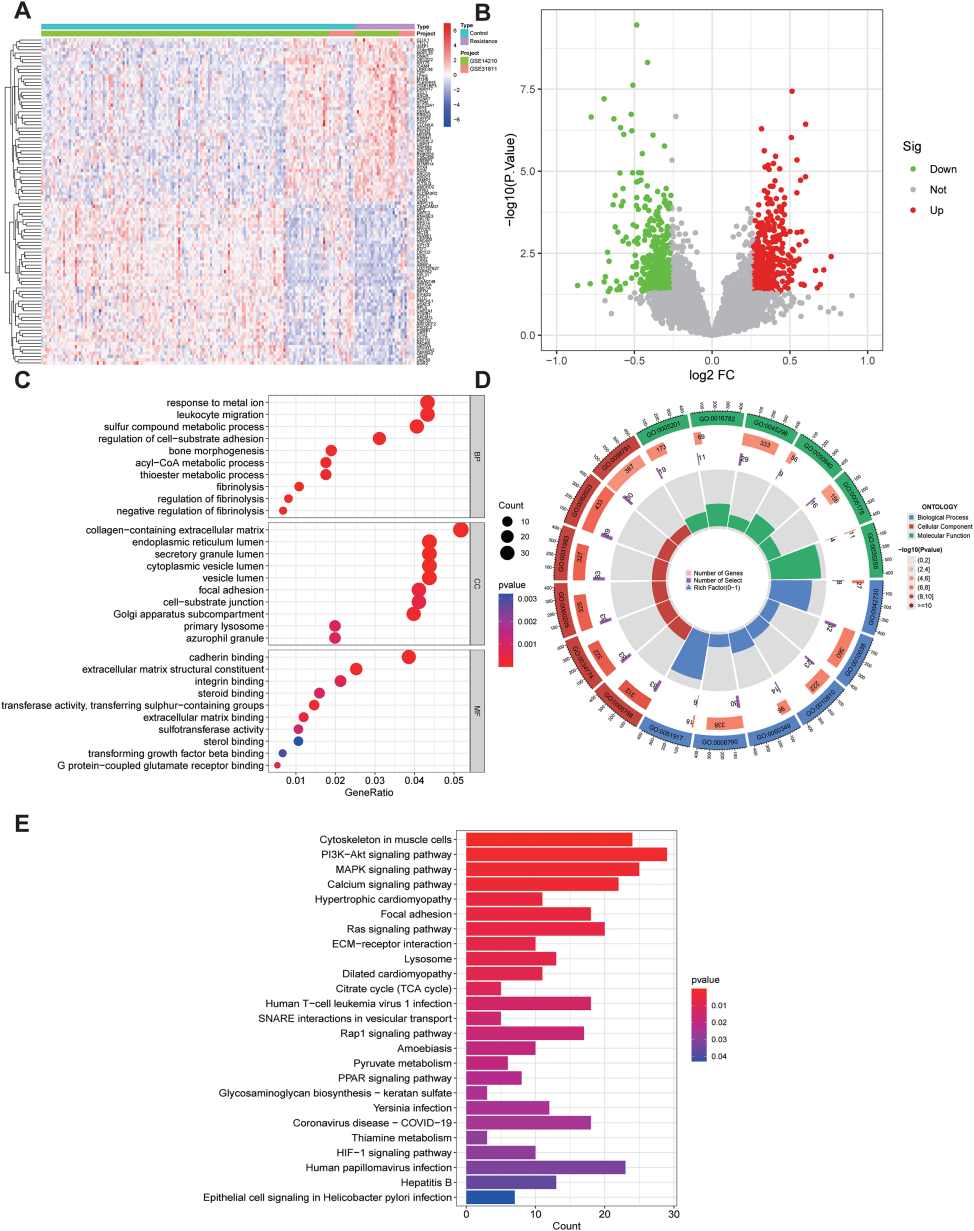
Identification of DEGs associated with chemoresistance in GC. **(A)** Heatmap showing the overall expression profiles of 827 DEGs (379 downregulated, 448 upregulated) in chemoresistant and chemosensitive samples from the GSE14210 and GSE31811 datasets. **(B)** Volcano plot showing the distribution and significance of DEGs. **(C)** Bubble plot of GO enrichment analysis of DEGs. **(D)** Circle plot of GO enrichment analysis of DEGs. **(E)** Bar chart of KEGG pathway enrichment analysis of DEGs.

A total of 827 DEGs were identified from the GSE14210 and GSE31811 transcriptome datasets, including 379 downregulated and 448 upregulated genes. Distinct expression patterns between chemoresistant and chemosensitive samples were observed, as illustrated by the heatmap (**Figure 2A**) and volcano plot (**Figure 2B**). GO enrichment analysis (**Figures 2C,D**) revealed that these DEGs were mainly involved in leukocyte migration, metal ion response, regulation of cell–matrix adhesion, osteogenesis, and sulfur compound metabolism in biological processes; localized to azurophilic granules, primary lysosomes, collagen-containing extracellular matrix, secretory vesicle lumen, and ER lumen in cellular components; and enriched in steroid binding, TGF-β binding, integrin binding, and extracellular matrix structural constituent in molecular functions. KEGG pathway analysis (**Figure 2E**) indicated that these genes participated in signaling pathways related to *Helicobacter pylori* infection, HIF-1, PPAR, ECM–receptor interaction, Rap1, Ras, MAPK, and PI3K–Akt, as well as lysosome, carbohydrate metabolism, and calcium signaling pathways, suggesting a potential synergistic role of metabolic reprogramming, inflammatory response, and signaling pathway remodeling in the development of GC chemoresistance.

### Refinement of core gene features associated with chemoresistance using machine learning

**Figure 3:**
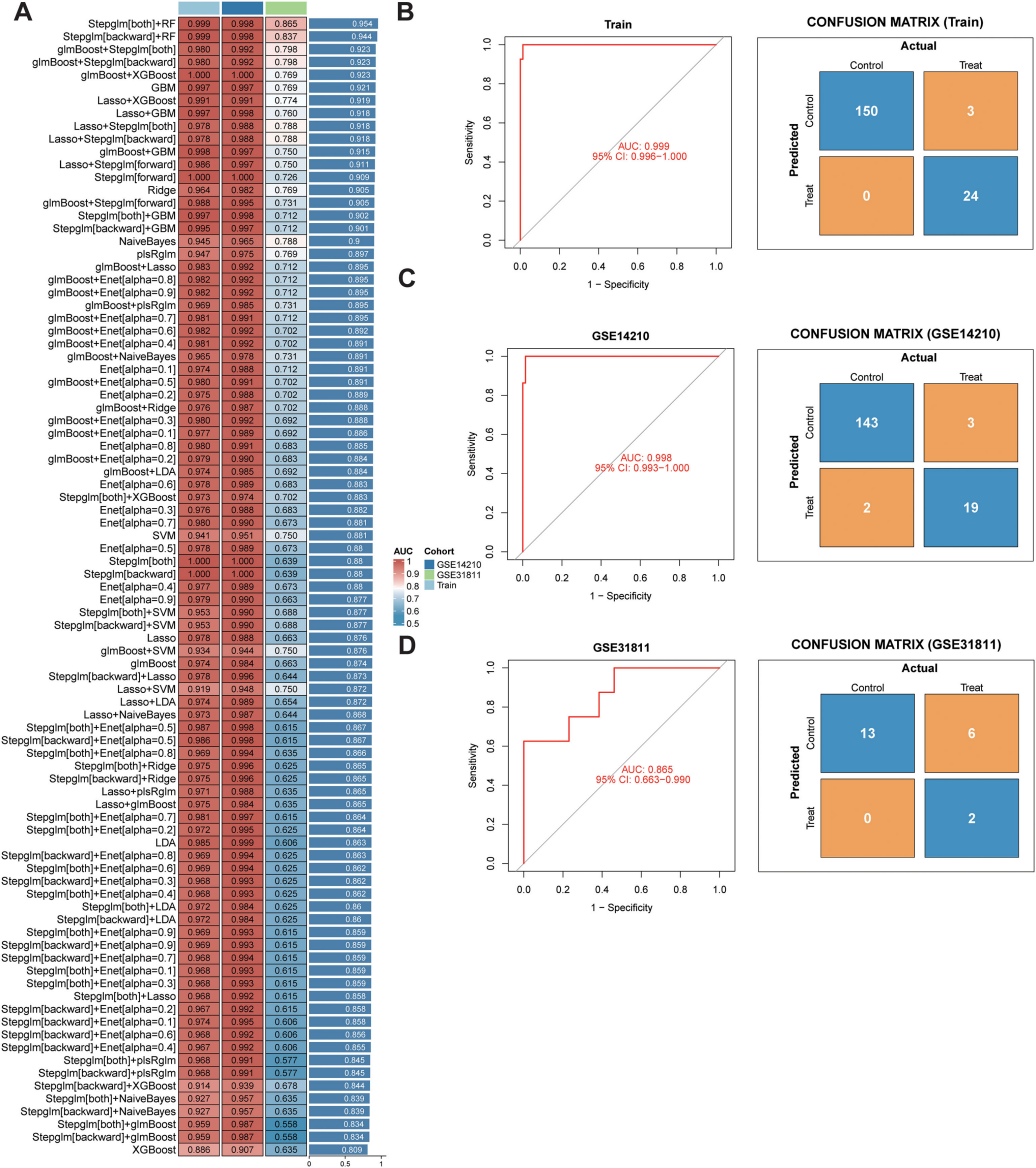
Machine-learning-based screening of core genes associated with drug resistance. **(A)** Performance comparison of 113 models. **(B–D)** Confusion matrices showing robust classification of chemoresistant and sensitive samples in training and validation cohorts.

A total of 113 machine learning model combinations were constructed to refine candidate genes associated with chemoresistance. As shown in **Figure 3A**, the Stepglm[both] + RF model achieved the best performance, with an average AUC of 0.954 in the training set and 0.865 in the validation set, outperforming other methods. Genes selected by this optimal model effectively discriminated chemoresistant from chemosensitive samples, as reflected by the corresponding confusion matrices (**Figures 3B–D**).

### Mendelian randomization analysis supports genetically informed associations between gastric cancer and key genes

**Figure 4:**
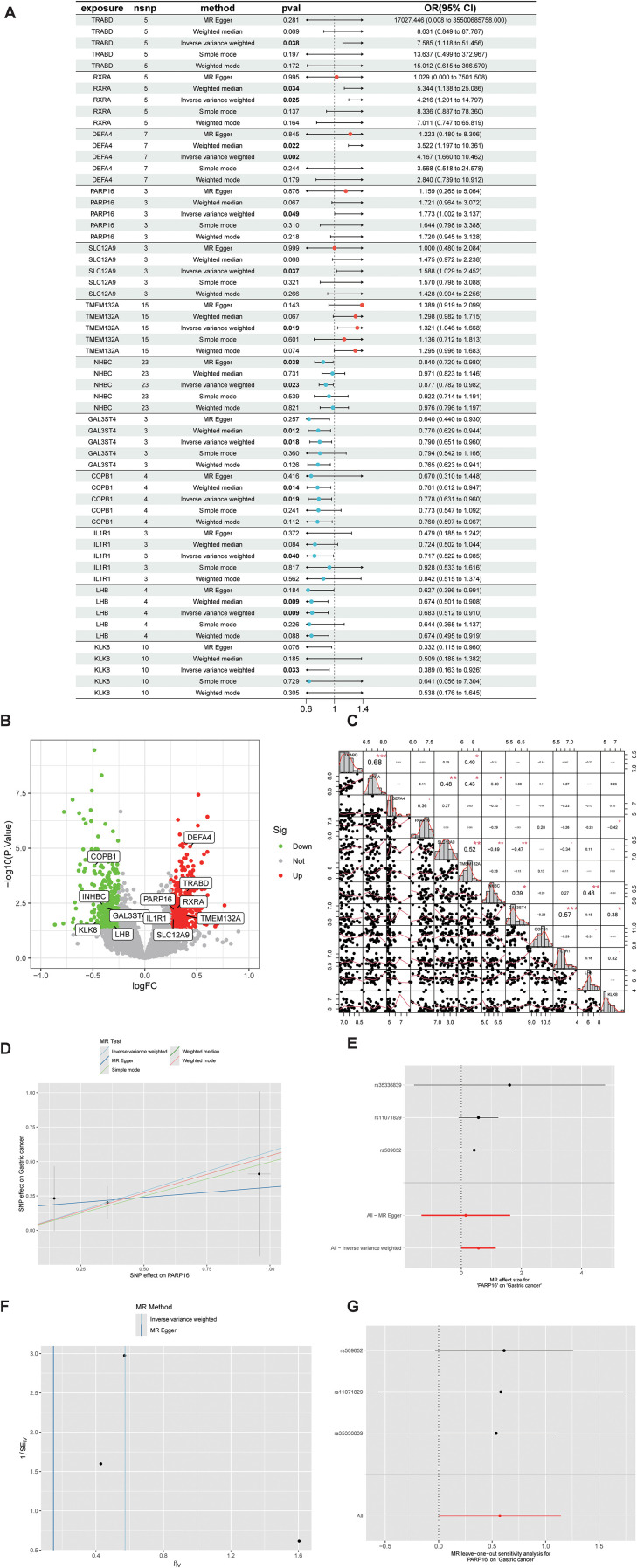
MR analysis of candidate genes. **(A)** Forest plot of inverse variance weighted (IVW) estimates showing the expression of risk-associated genes (TRABD, RXRA, DEFA4, PARP16, SLC12A9, an d TMEM132A) and protective genes (INHBC, GAL3ST4, COPB1, IL1R1, LHB, and KLK8). **(B)** Expression of risk-associated genes (TRABD, RXRA, DEFA4, PARP16, SLC12A9, and TMEM132A) and protective genes (INHBC, GAL3ST4, COPB1, IL1R1, LHB, and KLK8). **(C)** Correlation analysis of risk-associated genes (TRABD, RXRA, DEFA4, PARP16, SLC12A9, and TMEM132A) and protective genes (INHBC, GAL3ST4, COPB1, IL1R1, LHB, and KLK8). **(D–F)** Scatter plots, forest plots, and funnel plots of PARP16 demonstrate its causal effect on GC risk, assess the causal effect of individual SNPs, and assess overall heterogeneity, respectively. **(G)** Leave-one-out analysis further visualizes the independent effect of PARP16 on GC risk.

Based on public eQTL and GWAS data, systematic MR analysis was performed for the candidate genes. As shown in **Figure 4A**, several genes showed significant genetically supported associations with GC risk under the MR framework. TRABD, RXRA, DEFA4, PARP16, SLC12A9, and TMEM132A were identified as risk factors (OR > 1, *p* < 0.05), while INHBC, GAL3ST4, COPB1, IL1R1, LHB, and KLK8 served as protective factors (OR < 1, *p* < 0.05). Expression and correlation analyses further supported these findings with consistent trends (**Figures 4B,C**). The eQTL-based MR analysis suggests that the association of PARP16 with GC risk is linked to transcriptional regulation rather than protein-altering variants, as the instrumental SNPs primarily affect gene expression. This finding is corroborated by our *in vitro* experiments, where PARP16 mRNA was significantly elevated in resistant cells (approximately 3.1-fold, *p* < 0.01), suggesting that transcriptional dysregulation may contribute to the development of chemoresistance. *F* statistics were calculated to assess the strength of instrumental variables. MR-Egger intercept tests and Cochran’s *Q* statistics were used to evaluate horizontal pleiotropy and heterogeneity.

Among the validated genes, PARP16 was repeatedly identified as a risk factor in transcriptomic, machine learning, and MR analyses. The IVW analysis demonstrated a significant association between elevated PARP16 expression and GC risk (OR = 1.77, 95% CI = 1.00–3.14, *p* = 0.049). The direction of effect was consistent across MR-Egger, weighted median, and weighted mode methods (**Figures 4D–F**), with no significant heterogeneity or horizontal pleiotropy observed. Leave-one-out sensitivity analysis (**Figure 4G**) further demonstrated that removal of individual SNPs did not substantially alter the overall causal effect, supporting the robustness of the results. Therefore, PARP16 was established as a core candidate gene closely associated with GC chemoresistance.

### Molecular features of PARP16 and immune infiltration analysis

**Figure 5:**
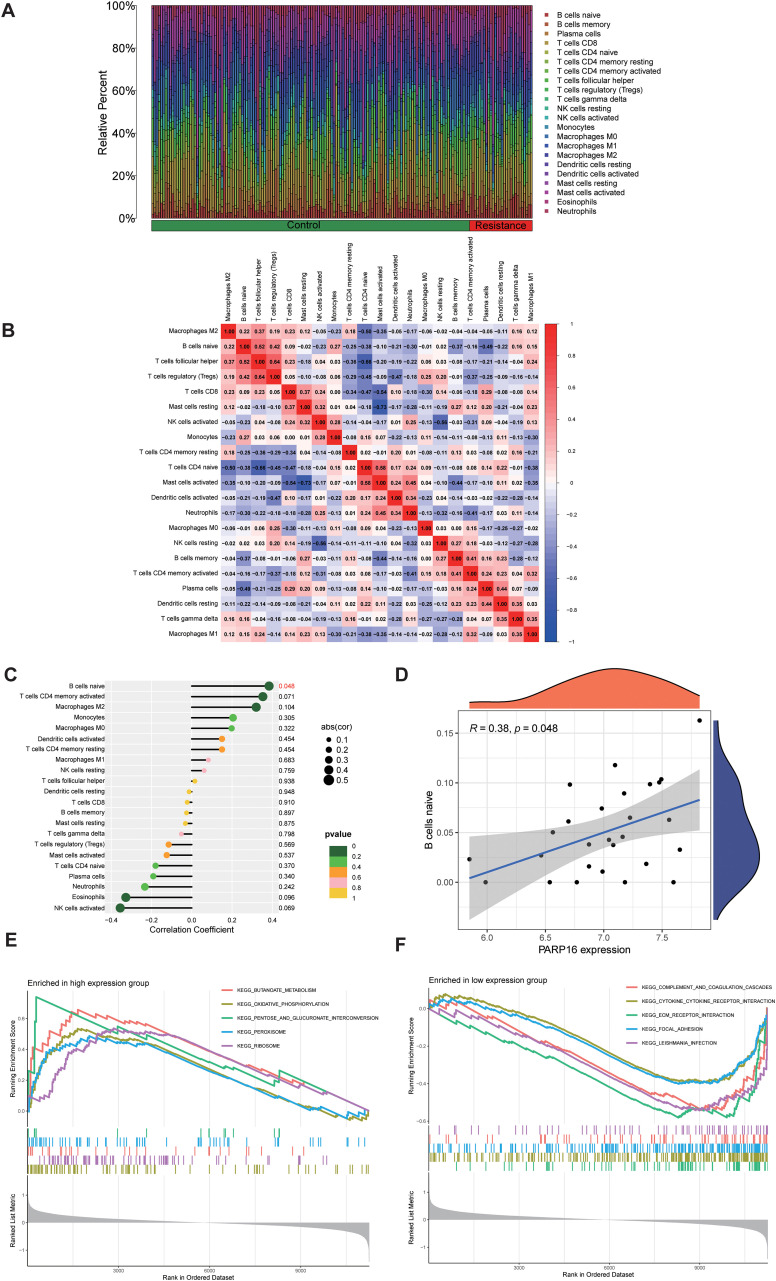
Molecular signatures of PARP16 and immune infiltration analysis. **(A)** Stacked plot of immune cell composition. **(B)** Heatmap of immune cell correlation analysis. **(C)** Correlation analysis between PARP16 and immune cell infiltration. **(D)** Scatter plot showing the positive correlation between PARP16 and naive B cells (*R* = 0.38, *p* = 0.048). **(E)** GSEA enrichment analysis of the PARP16 high-expression group. **(F)** GSEA enrichment analysis of the PARP16 low-expression group.

CIBERSORT-based immune infiltration analysis further revealed the relationship between PARP16 and the immune microenvironment. The stacked bar plot of immune cell composition showed clear differences in immune cell subpopulation proportions across tumor samples (**Figure 5A**), while the immune cell correlation heatmap revealed patterns of interaction between immune cells (**Figure 5B**). Correlation analysis showed that PARP16 expression was significantly associated with multiple immune cell infiltration levels (**Figure 5C**), with a notable positive correlation with naive B cells (*R* = 0.38, *p* = 0.048, **Figure 5D**). These results suggest that PARP16 may be involved not only in metabolism and stress adaptation but also in alterations in the immune landscape associated with chemoresistance in GC.

To further explore the potential mechanisms of PARP16, patients were stratified into high- and low-expression groups, followed by GSEA. Results showed that the high-expression group of PARP16 was significantly enriched in oxidative phosphorylation, peroxisome, butanoate metabolism, pentose and glucuronate interconversions, and ribosome pathways (**Figure 5E**), indicating a role in promoting energy metabolism and protein synthesis. Conversely, the low-expression group was enriched in complement and coagulation cascades, cytokine–cytokine receptor interaction, ECM–receptor interaction, focal adhesion, and infection-related pathways (**Figure 5F**), suggesting that downregulation of PARP16 may be linked to the activation of immune and inflammation-related responses.

### Single-cell transcriptome analysis reveals cell-specific expression of PARP16

**Figure 6:**
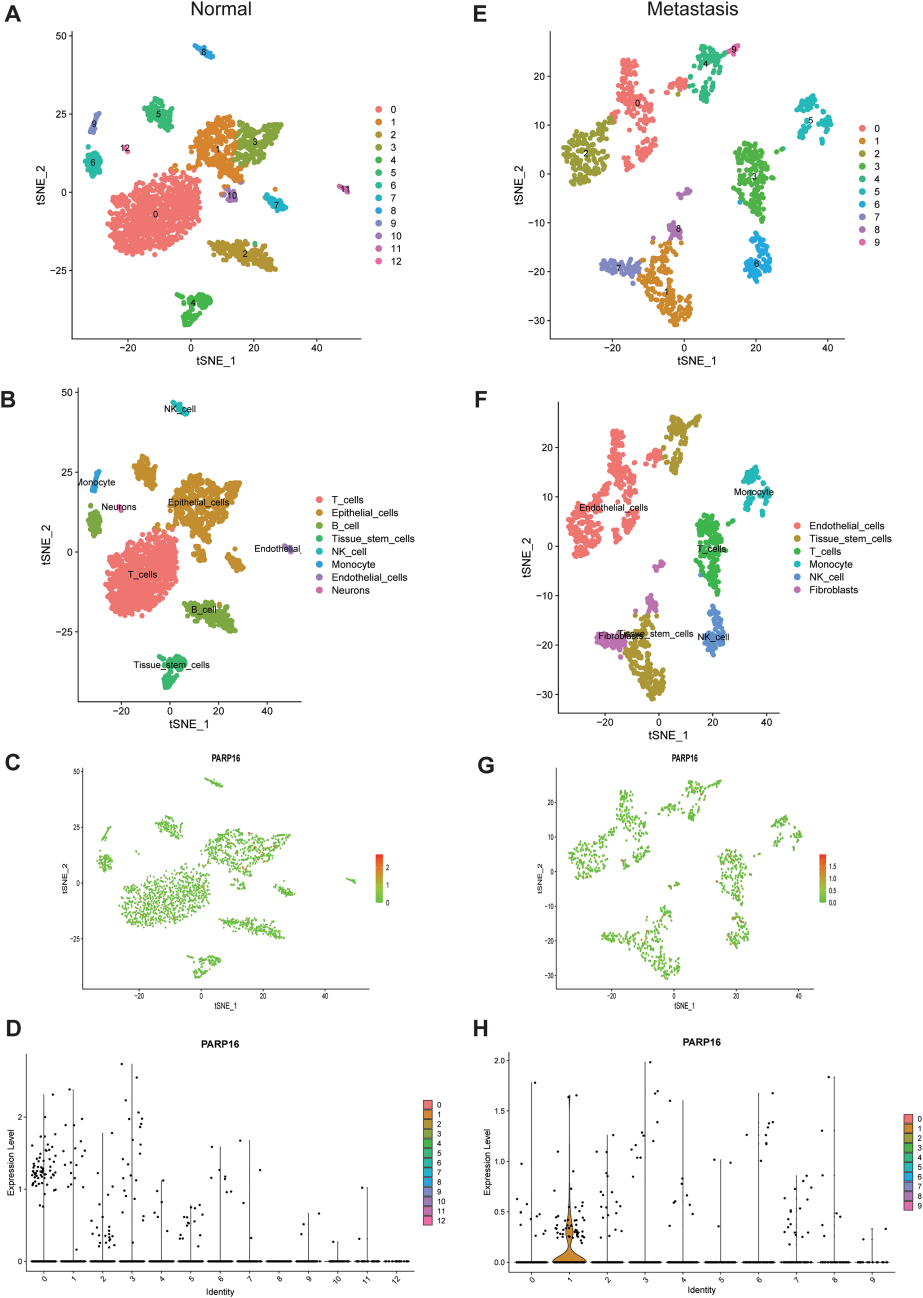
Single-cell transcriptome analysis of PARP16 in GC. **(A)** UMAP clustering results of single-cell data from normal gastric tissue. **(B)** Cell-type annotation of normal gastric tissue. **(C)** Scatter plot of PARP16 expression in single cells of normal gastric tissue. **(D)** Violin plot of PARP16 expression in different cell types of normal gastric tissue. **(E)** UMAP clustering results of single-cell data from GC tissue. **(F)** Cell-type annotation of GC tissue. **(G)** Scatter plot of PARP16 expression in single cells of GC tissue. **(H)** Violin plot of PARP16 expression in different cell types of GC tissue.

Single-cell transcriptomic analysis of the GSE183904 dataset was performed using UMAP-based clustering and cell-type annotation in both GC and normal gastric tissues. Results demonstrated that PARP16 expression was significantly lower in normal gastric tissue, being detectable only in a subset of epithelial cells (**Figures 6A–D**). In contrast, PARP16 was predominantly highly expressed in the tissue stem cell population of GC tissue (**Figures 6E–H**).

### Upregulation of PARP16 and activation of the UPR pathway in NCI-N87/DDP cells

**Figure 7:**
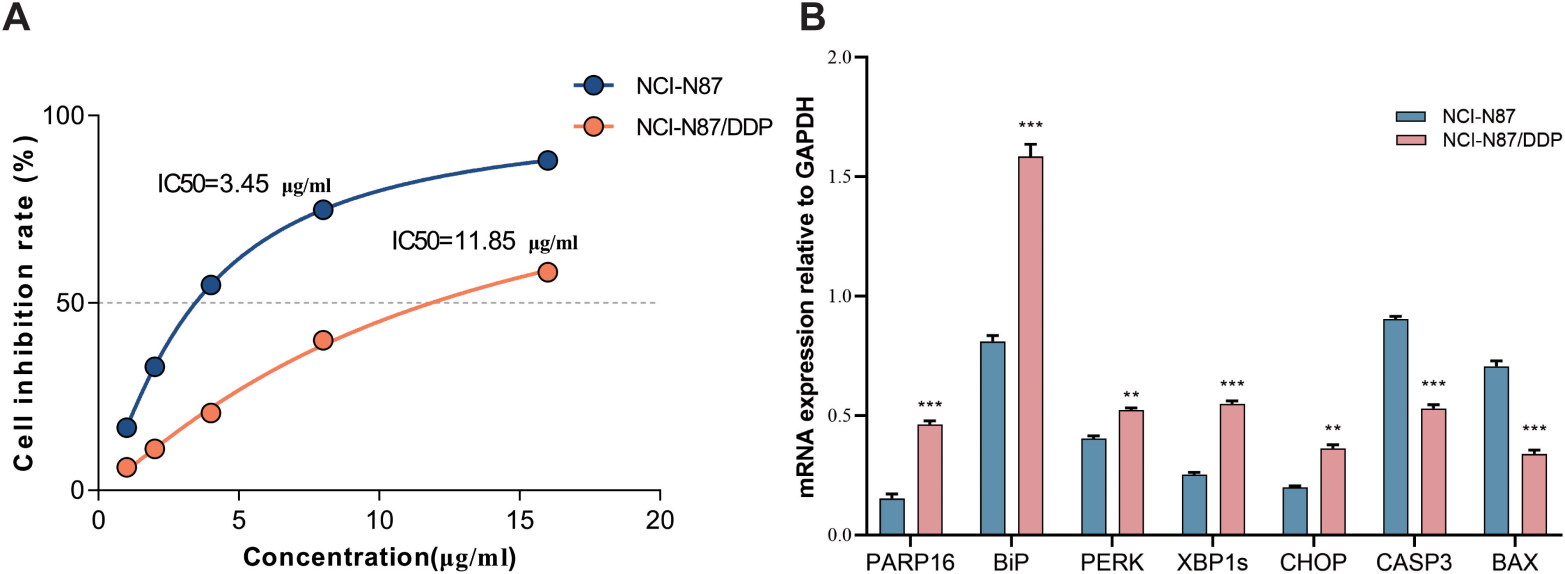
Upregulation of PARP16 in the NCI-N87/DDP cell line and activation of the UPR pathway. **(A)** Dose–response curve shows that the IC_50_ of NCI-N87/DDP (11.85 μg/mL) is higher than that of the parental cells (3.45 μg/mL). **(B)** qRT-PCR analysis shows changes in PARP16, BiP, PERK, XBP1s, CHOP, CASP3, and BAX expression in NCI-N87/DDP cells compared to parental NCI-N87 cells. Compared to the control group, ***p < 0.05, **p < 0.01, *p < 0.001.

*In vitro* experiments successfully established the cisplatin-resistant cell line NCI-N87/DDP through long-term exposure to gradually increasing cisplatin concentrations. Drug sensitivity assays confirmed a clear shift in cisplatin response between parental and resistant cells. NCI-N87/DDP exhibited an IC_50_ of 11.82 ± 1.07 μg/mL, significantly higher than that of parental NCI-N87 cells (3.45 ± 0.41 μg/mL), corresponding to a resistance index of approximately 3.43 (*p* < 0.001, **Figure 7A**), indicating enhanced survival under cisplatin treatment. qRT-PCR analysis further revealed that PARP16 expression was significantly elevated in resistant cells (approximately 3.1-fold, *p* < 0.01), accompanied by upregulation of UPR-related markers (BiP, PERK, XBP1s, and CHOP) and downregulation of apoptosis-related factors CASP3 and BAX (**Figure 7B**). These findings suggest that elevated PARP16 expression is associated with the activation of adaptive UPR signaling and reduced susceptibility to cisplatin-induced apoptosis.

### Inhibition of the PARP16–UPR axis reverses cisplatin resistance

**Figure 8:**
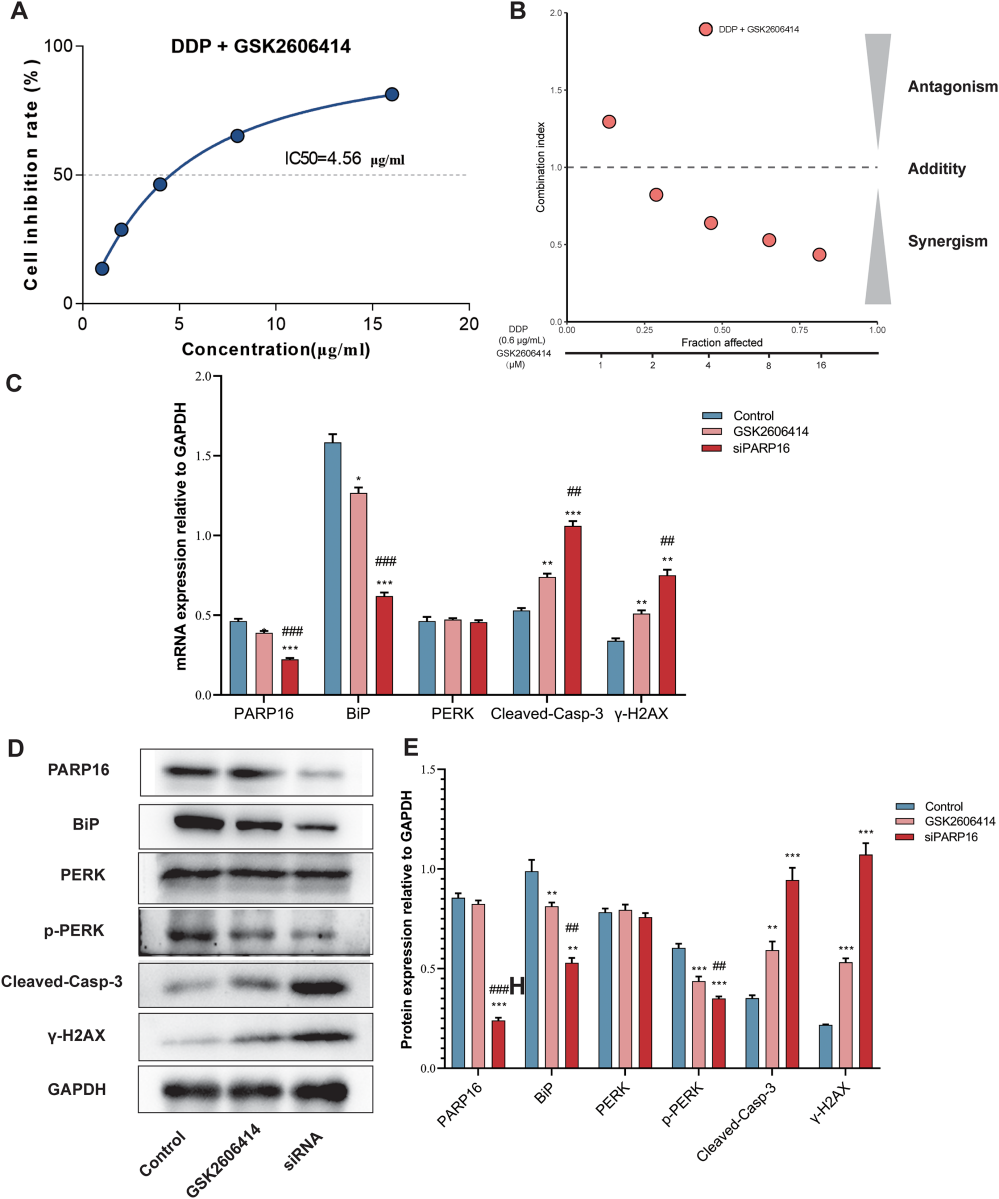
Inhibition of the PARP16–UPR axis reverses cisplatin resistance. **(A)** Cell viability assay shows cell survival after the PERK inhibitor GSK2606414 is combined with cisplatin. **(B)** Combination index (CI) analysis demonstrates synergistic effects. **(C)** RT-qPCR validation of relevant mRNA expression after GSK2606414 treatment or PARP16 knockdown. **(D)** Western blot validation of relevant protein changes after GSK2606414 treatment or PARP16 knockdown. Compared with the control group, ***p < 0.05, **p < 0.01, *p < 0.001.; compared with the GSK2606414 group, #p < 0.05, ##p < 0.01, ###p < 0.001. Quantification represents densitometric analysis from three independent experiments, normalized to GAPDH.

To further validate the role of the PARP16–UPR axis in cisplatin resistance, NCI-N87/DDP cells were treated with the PERK inhibitor GSK2606414 in combination with cisplatin. Results demonstrated that the combination significantly reduced cell viability, with the IC_50_ of resistant cells decreasing to 4.67 μg/mL, approaching the level of parental NCI-N87 cells (**Figure 8A**). CI analysis indicated that most combinations had CI < 0.8, suggesting strong synergistic effects (**Figure 8B**).

At the transcriptional level, resistant cells exhibited increased expression of PARP16 and BiP, accompanied by reduced expression of cleaved caspase-3 and γ-H2AX. Upon treatment with GSK2606414 or siPARP16 knockdown, PARP16 and BiP expression decreased, p-PERK levels were markedly reduced, and cleaved caspase-3 and γ-H2AX expression were restored (**Figure 8C**). Consistent changes were observed at the protein level. PARP16 and BiP decreased under siRNA conditions, while p-PERK was reduced after both GSK2606414 and siRNA treatment. cleaved caspase-3 and γ-H2AX levels were elevated accordingly (**Figures 8D,E**).

To assess the generalizability of these findings, parallel experiments were performed in an independent cisplatin-resistant GC cell line, AGS/DDP. Combined treatment with GSK2606414 and cisplatin similarly decreased cell viability and enhanced cisplatin sensitivity in AGS/DDP cells. Molecular analyses revealed expression patterns consistent with those observed in NCI-N87/DDP cells, characterized by downregulation of PARP16, BiP, and p-PERK and upregulation of cleaved caspase-3 and γ-H2AX following inhibition of the PARP16–UPR axis (**Supplementary Figure 1**).

## Discussion

GC is one of the most prevalent and lethal malignancies of the digestive system worldwide (38, 39). Cisplatin remains a frontline chemotherapeutic agent (40); however, most patients gradually develop resistance during treatment, which is a major cause of reduced efficacy and poor prognosis (41, 42). Previous studies have shown that the mechanisms of GC chemoresistance are complex and involve enhanced DNA repair, overactivation of drug efflux pumps, metabolic reprogramming, suppression of apoptosis, and remodeling of the tumor microenvironment (43–45). In this study, by applying an integrative multi-level analytical framework combining transcriptomics, machine learning, MR, and single-cell validation, we prioritized PARP16 as a key candidate associated with cisplatin resistance.

A central finding of this study is the association between PARP16 expression and activation of ER stress-associated UPR. PARP16 is an ER-resident mono-ADP-ribosyltransferase primarily involved in the regulation of ER stress and UPR (46). In multiple cancer types, the UPR has been recognized as a crucial survival mechanism that enables tumor cells to withstand adverse microenvironments and chemotherapy stress (47–49). Our results showed that UPR markers such as BiP, p-PERK, XBP1s, and CHOP were significantly upregulated in resistant cell lines, whereas apoptosis-related factors were downregulated. These findings indicate that elevated PARP16 expression is associated with adaptive UPR activation and reduced susceptibility to splatting-induced apoptosis in resistant GC cells. This is consistent with previous reports in breast and liver cancers where UPR promoted tumor survival and resistance (50, 51). Overall, these observations support a model in which PARP16 contributes to cisplatin resistance in GC by sustaining adaptive UPR signaling and weakening apoptosis under chemotherapy-induced stress.

PARP16 was consistently identified as a resistance-associated factor across multiple analyses. It was repeatedly highlighted through differential expression analysis, machine learning-based feature selection, and MR analysis, suggesting that its relevance to chemoresistance is not restricted to a single methodological approach. Machine learning can markedly improve the accuracy of predicting tumor drug responses and provide tools for clinical risk stratification and personalized treatment (52, 53). MR uses genetic variants as instrumental variables to strengthen causal inference under specific assumptions and reduce confounding and reverse causation relative to conventional observational analyses (54, 55). Unlike traditional observational studies, MR can effectively reduce confounding bias and reverse causation, providing genetically anchored evidence that may support causal inference. Notably, the MR analysis was based on eQTLs, indicating that transcriptional regulation of PARP16, rather than protein-altering variants, underlies the observed associations with GC risk. When considered together with functional validation in resistant cell models, these results suggest that increased PARP16 expression may contribute to chemoresistance-related phenotypes in GC.

Pathway enrichment analyses also provided contextual support for the resistance observed in this study. Multi-omics analysis indicated that the DEGs were significantly enriched in classical resistance-related pathways, including PI3K–Akt, MAPK, and ECM–receptor interactions. These pathways have been extensively documented in previous studies as being closely related to resistance of tumor cells to various chemotherapeutic agents. The PI3K–Akt pathway enhances cell survival under chemotherapy stress by promoting DNA repair and inhibiting apoptosis (56). The MAPK pathway is frequently linked to the activation of drug efflux pumps and the regulation of the cell cycle (57). ECM–receptor interactions not only mediate adhesion between tumor cells and the extracellular matrix but also strengthen survival and migration through the integrin–FAK axis, forming a crucial foundation of resistance (58–60). These findings are highly consistent with earlier reports and further support the reliability of our analytical approach.

Beyond stress signaling, our analyses suggest that PARP16 expression may also be associated with broader metabolic and microenvironmental features. GSEA revealed that high PARP16 expression was associated with activation of oxidative phosphorylation, peroxisome, and ribosome pathways, whereas low expression was enriched in complement and coagulation cascades, cytokine–cytokine receptor interactions, ECM–receptor interactions, and focal adhesion pathways. In parallel, CIBERSORT-based immune infiltration analysis further showed that PARP16 expression was significantly positively correlated with naive B cells. These findings indicate that inhibition of the PARP16–UPR axis may suppress adaptive UPR activation, restore immune–inflammatory and ECM-related responses, and consequently enhance DNA damage and apoptosis signals to reverse cisplatin resistance. This also suggests that high PARP16 expression may endow GC stem-like cells with greater stress adaptation and resistance capacity, while its limited expression in normal tissues supports its potential as a therapeutic target with a favorable safety window. Single-cell sequencing further revealed that PARP16 was enriched in tissue stem cell populations of GC tissues, while its expression was low in normal tissues. Cancer stem-like cells are widely recognized as seeds of chemoresistance and relapse, as they can maintain DNA repair capacity, adapt to stress, and evade apoptosis for long-term survival. PARP16 may therefore serve as a molecular link between stemness and resistance, with selective expression making it an attractive target.

In functional experiments, inhibition of the UPR pathway significantly reduced the IC_50_ of resistant cells and restored DNA damage and apoptosis signaling, indicating that the resistant phenotype is partially dependent on sustained stress-adaptive responses. This is consistent with previous findings that UPR inhibitors can enhance chemosensitivity. More importantly, combination treatment with UPR inhibitors and cisplatin exhibited strong synergistic effects, suggesting that targeting the PARP16–UPR axis may represent an effective strategy for overcoming cisplatin resistance. From a clinical perspective, PARP16 holds dual potential: as a predictive biomarker of chemoresistance and as a therapeutic target. Unlike broad DNA repair factors and other classical resistance-related molecules, the low expression of PARP16 in normal tissues offers a more favorable safety profile for drug development. With the continuous emergence of UPR inhibitors, future strategies may include PARP16-targeted drugs or combinations of UPR inhibitors with cisplatin, and even triple regimens integrating stemness pathway inhibitors, to achieve better outcomes in resistant patients. Overall, these results suggest that cisplatin resistance can, to a certain extent, depend on the plastic stress adaptation process. Intervening in the PARP16–UPR axis may provide a potential therapeutic window for restoring chemotherapy sensitivity. Although the relevant conclusions still need further verification, this study provides a theoretical basis for the treatment approach of combining stress regulation strategies with traditional chemotherapy.

This study has several limitations. The bioinformatic analyses relied primarily on publicly available transcriptomic and single-cell datasets, and sample heterogeneity as well as potential batch effects may influence the robustness of the findings. In addition, the analytical framework adopted here integrates multiple data layers in a stepwise manner rather than through formal joint multi-omics modeling. Future studies incorporating network-based or Bayesian integrative approaches may further refine the relationships among these molecular features. The experimental validation, although supportive, remains limited. Functional experiments were conducted in two cisplatin-resistant GC cell lines, which provides initial evidence for reproducibility across models. However, *in vivo* validation and patient-derived systems were not included, which restricts the translational generalizability of the findings. Direct baseline protein comparisons between parental and resistant cells were also not systematically evaluated in all models and warrant further investigation. More definitive genetic perturbation approaches, such as CRISPR-mediated knockout of PARP16, would further strengthen causal interpretation at the cellular level. From a mechanistic perspective, the UPR represents a highly dynamic and branched signaling network, and PARP16 is unlikely to function as the sole regulator. The present study mainly focused on the PERK branch of the UPR pathway, whereas the potential contributions of the ATF6 and IRE1 branches remain to be explored. In addition, interactions between PARP16 and other resistance-associated pathways, including PI3K–Akt, MAPK, and NF-κB signaling, require further investigation. Methodological limitations related to the MR analysis should also be considered. The eQTL datasets used to derive instrumental variables were obtained primarily from population-level non-tumor tissues, and therefore may not fully capture tumor-specific transcriptional regulation in GC. Although the MR results provide genetically supported evidence linking PARP16 expression to chemoresistance-related phenotypes, they do not account for potential coding variants that may alter PARP16 protein function. Integrating both eQTL and protein quantitative trait locus (pQTL) datasets in future analyses may provide a more comprehensive understanding of PARP16 regulation at transcriptional and post-translational levels. Finally, although our findings suggest that the PARP16–UPR axis may represent a potential therapeutic target, highly selective PARP16 inhibitors remain underdeveloped. Further pharmacological studies will therefore be required to evaluate the clinical feasibility of targeting this pathway for overcoming chemoresistance in GC.

## Conclusion

This study identified PARP16 as a key candidate associated with cisplatin resistance in GC through an integrative multi-level analytical framework combined with cellular validation. PARP16 was enriched in resistant cells and tumor stem-like populations and was associated with activation of ER stress/UPR signaling. Inhibition of the PARP16–UPR axis restored DNA damage and apoptotic signaling, supporting its potential relevance as a biomarker and therapeutic target in chemoresistant GC. Blocking the PARP16–UPR axis restored DNA damage accumulation and apoptotic signaling, thereby reversing the drug-resistant phenotype. This discovery not only deepens our understanding of the mechanisms of drug resistance in GC but also suggests that PARP16 may serve as a biomarker for predicting drug resistance and a potential therapeutic target, providing new insights into clinical approaches to reversing drug resistance and optimizing chemotherapy regimens.

## Data Availability

The datasets analyzed in this study are publicly available. Transcriptomic and single-cell datasets were obtained from the Gene Expression Omnibus (GEO) under accession numbers GSE14210, GSE31811, and GSE183904. Public eQTL summary statistics were obtained from the GTEx and eQTLGen resources, and GWAS summary statistics were obtained from FinnGen and the IEU OpenGWAS database. Further details are provided in the article and **Supplementary Material**.

## Supplementary material

Supplementary Figure 1 Inhibition of the PARP16-UPR axis reverses cisplatin resistance. Cell viability assay shows cell survival after the PERK inhibitor GSK2606414 is combined with cisplatin. **(B)** RT-qPCR validation of relevant mRNA expression after GSK2606414 treatment or PARP16 knockdown. **(C)** Western blot validation of relevant protein changes after GSK2606414 treatment or PARP16 knockdown. Compared with the control group, *<0.05, **<0.01, ***<0.001; compared with the GSK2606414 group, #<0.05, ##<0.01, ###<0.001. Quantification represents densitometric analysis from three independent experiments, normalized to GAPDH.

Supplementary Figure 2 Whole, uncropped Western blot. The original, uncropped membrane is shown for PARP16, BiP, PERK, p-PERK, cleaved caspase-3, γ-H2AX, and GAPDH.
