# Supplementary material for: Multi-omics integration and Mendelian randomization elucidate the PARP16–UPR axis driving chemoresistancein gastric cancer
Source: Front Oncol. 2026 May 1;16:1785100. doi: 10.3389/fonc.2026.1785100 (PMC13175845; doi:10.3389/fonc.2026.1785100)
Supplement: Supplementary Figure 1 — Inhibition of the PARP16-UPR axis reverses cisplatin resistance. Cell viability assay shows cell survival after the PERK inhibitor GSK2606414 is combined with cisplatin. (B) RT-qPCR validation of relevant mRNA expression after GSK2606414 treatment or PARP16 knockdown. (C) Western blot validation of relevant protein changes after GSK2606414 treatment or PARP16 knockdown. Compared with the control group, *<0.05, **<0.01, ***<0.001; compared with the GSK2606414 group, #<0.05, ##<0.01, ###<0.001. Quantification represents densitometric analysis from three independent experiments, normalized to GAPDH. [file DataSheet1.pdf]

PARP16

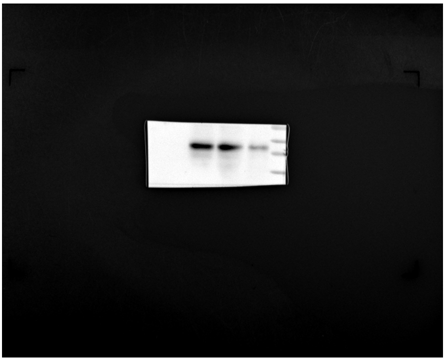

Control GSK2606414 siRNA

BiP

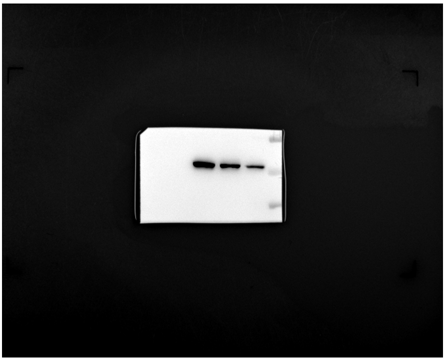

Control GSK2606414 siRNA

PERK

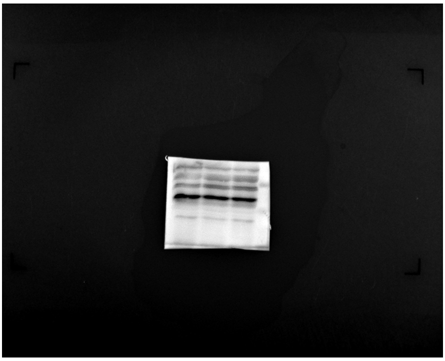

Control GSK2606414 siRNA

p-PERK

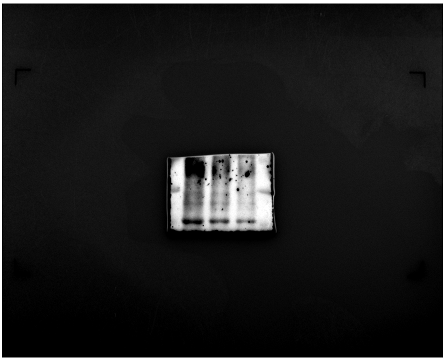

Control GSK2606414 siRNA

Cleaved-Casp-3

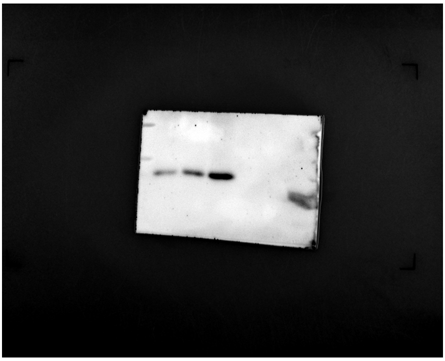

Control GSK2606414 siRNA

γ-H2AX

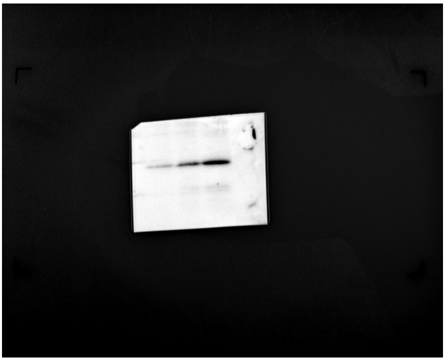

Control GSK2606414 siRNA

GAPDH

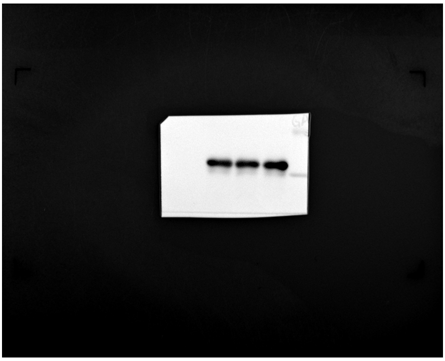

Control GSK2606414 siRNA
