# Supplementary figures and images for: Multi-omics integration and Mendelian randomization elucidate the PARP16–UPR axis driving chemoresistancein gastric cancer
Source: Front Oncol. 2026 May 1;16:1785100. doi: 10.3389/fonc.2026.1785100 (PMC13175845; doi:10.3389/fonc.2026.1785100)

Control

GSK2606414

siRNA

PARP16

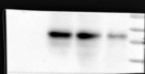

BiP

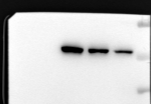

PERK

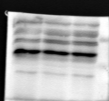

p-PERK

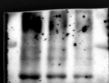

Activated-Casp-3

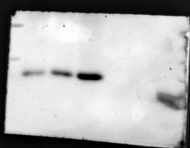

$\gamma$ -H2AX

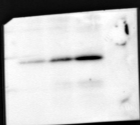

GAPDH

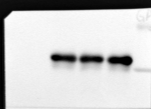

Supplement: Supplementary Figure 2 — Whole, uncropped Western blot. The original, uncropped membrane is shown for PARP16, BiP, PERK, p-PERK, cleaved caspase-3, γ-H2AX, and GAPDH. [file DataSheet2.pdf]
